# Surgery in *Staphylococcus aureus* Infective Endocarditis: *Clinical Outcomes, Neurological Sequelae, and Prognostic Implications*

**DOI:** 10.3390/jcm14197114

**Published:** 2025-10-09

**Authors:** Ahmed Elderia, Julian Hinzmann, Patricia Soehne, Walid Bennour, Thorsten Wahlers, Carolyn Weber

**Affiliations:** 1 Department of Cardiac Surgery, Heart Center, University of Cologne, D-50937 Cologne, Germany; 2 Department of Cardiology, Pneumology, Angiology and Intensive Care Medicine, Heart Center, University of Cologne, D-50937 Cologne, Germany

**Keywords:** *Staphylococcus aureus*, infective endocarditis, embolic, cerebrovascular

## Abstract

**Background:***Staphylococcus aureus* infective endocarditis (SA-IE) is believed to provoke higher rates of complications compared to other organisms that commonly cause IE (non-SA-IE). We believe that *Staphylococcus aureus* (*S. aureus*) has a high propensity to cause embolic events and cerebrovascular neurological complications. **Methods:** We conducted a single-center retrospective analysis, encompassing 529 patients who had undergone valve surgery for IE. Patients were divided according to causative microorganism into SA-IE and non-SA-IE groups. Subsequently, analyses of outcome differences between the two groups were performed, with a focus on neurological complications. **Results:** In the examined population, 128 (24.2%) had IE mediated by *S. aureus*. Patients with SA-IE were mostly male (69.3%) but had a higher proportion of females compared to non-SA-IE patients (30.7% vs. 21.8%; *p* = 0.039) and were significantly younger (61.1 [45.8–69.9] vs. 66.1 [54.3–74.4]; *p* = 0.002). Patients with SA-IE had comparable comorbidities to patients with non-SA-IE. Neurological complications were much more common in SA-IE (42.2%) compared to non-SA-IE (22.9%); (*p* < 0.001). Postoperative neurological complications were nearly equal in both groups—SA-IE 5.5% vs 6.2% in non-SA-IE (*p* = 0.752); 30-day mortality was significantly higher in patients with SA-IE vs. non-SA-IE (20.3% vs 12.5%; *p* = 0.028). However, the 1-year mortality rate did not differ between groups (29.4% vs. 22.2%; *p* = 0.121). **Conclusions:** Patients with SA-IE are subject to a higher incidence of neurological events prior to surgery and almost twice the short-term mortality rate compared to IE caused by other microorganisms. However, no observable discrepancy in the incidence of neurological events was found between SA- and non-SA-IE cases post-surgery.

## 1. Introduction

Infective endocarditis (IE) is a rare but serious disease. Despite improved diagnostic and therapeutic options, it continues to be a disease with many complications and high mortality. Over the past few years, there has been a change in the epidemiology of the patient clientele, with patients today getting older and increasingly suffering from cardiac preexisting conditions [1]. Worldwide, *Staphylococcus aureus* (*S. aureus*) is increasingly becoming the dominant causative agent in IE in up to 43% of cases [2]. *Staphylococcus aureus* infective endocarditis (SA-IE) tends to have a worse prognosis with more intense valve destruction compared with other microorganisms. This is due to its pathogenetic factors, for instance its ability to build up biofilm or to modify the immune system [3]. *S. aureus* infection is often linked to hospital stays and invasive procedures and poses an increasing risk of acquiring resistance, making treatment difficult [4].

In addition, IE caused by *S. aureus* has been found to be associated with a higher likelihood of neurological complications, but the impact of surgery on neurological complications is not yet fully understood [1]. The guideline recommendation is to perform surgery early in patients with a high risk for embolic complications. A neurological complication is not a strict contraindication for surgery, except if a very poor neurological outcome is likely [1]. Much information on the diagnostics, treatment, and prevention of SA-IE has been based on expert opinion [5,6].

Our aim is to (i) describe the clinical manifestation of *S. aureus* IE in surgical patients, (ii) analyze survival of patients with SA-IE and compare postoperative outcomes with IE caused by other pathogens, and (iii) gain insights into the relevance of surgery in patients with SA-IE and neurological complications. Thereby, we hope to gain knowledge in order to identify these patients at an early stage and to provide an appropriate treatment plan in the future.

## 2. Materials and Methods

### 2.1. Data Collection

We analyzed data from all IE patients who underwent cardiac surgery at our center between January 2009 and May 2020. The data we collected contain demographics, preexisting conditions, risk factors, symptoms, microbiological information, diagnostic information, surgery-related data, post-surgery complications, and long-term outcome including neurological complications. Follow-up was obtained by a review of hospital medical records and a review of the patient or the patient’s physician. The follow-up time for survival was measured from the date of operation to either the date of death or the date of the last contact. The study protocol was approved by the institutional review board (Ethics Committee of the Medical Faculty, University of Cologne, 17-407). Individual informed consent was waived due to the retrospective nature of the collected data.

### 2.2. Parameter Definition

SA-IE was defined when at least one of the following samples were positive for *S. aureus*: blood culture, a culture of valve material that was extracted during surgery, or PCR test for an intraoperative swap of the IE-suspected valve. In this analysis, we did not differentiate between Methicillin-sensible *S. aureus* (MSSA) or Methicillin-resistant *S. aureus* (MRSA) due to small number of MRSA-mediated IE. Sepsis was defined as an acute onset of at least one or more organ dysfunction in relation to infection. Preoperative neurological complications were defined as all stroke-related symptoms that were timely associated with the IE before cardiac surgery. Postoperative neurological complications were defined as all stroke-related symptoms which did occur postoperatively during the hospital stay. Follow-up neurological complications were defined as all stroke-related clinical symptoms which were reported in the time between the discharge from the reference hospital and the follow-up. We defined TIA as transient ischemia transient neurological symptoms and no sign of acute infarction [7]. Cerebral ischemia was defined as a stroke due to an embolic event diagnosed with cerebral imaging, and hemorrhagic stroke as new bleeding which was diagnosed via MRI or CT scan.

### 2.3. Surgery

The indication for surgery was justified with the criteria from the current ESC guidelines [6,8]. Surgery was performed by the attending cardiac surgeon, who also decided on the extent of the procedure. The extent of surgery was dependent on preoperative findings or intraoperative transesophageal echocardiography, which is performed as standard on all patients. The surgical procedure has already been described [9,10]. Vital signs were continuously monitored by the attending anesthetist who administered catecholamines, blood transfusion, and further medication at their and the surgeon’s discretion [9].

### 2.4. Statistical Analysis

For statistical analysis, we used IBM SPSS Statistics for Mac, Version 28.0 (IBM Corp., Armonk, NY, USA) [11]. Patient characteristics and preoperative factors were described using means ± standard deviation (SD), median and interquartile range [(IQR)], or frequencies and percentages as indicated. Depending on the data distribution, group differences were compared with the unpaired *t* test, Mann–Whitney U test, chi-square test, or Fisher’s exact test. All *p*-values reported are two-sided and considered statistically significant if inferior or equal to 5%. Survival estimation was performed with the Kaplan–Meier method. The log-rank test was used to assess differences in long-term mortality between SA-IE and non-SA-IE. Additionally, logistic regression was performed to evaluate the association between staphylococcal infection and 30-day mortality, and Cox regression was used to analyze 1-year mortality risk. The findings are documented as odds ratio (OR) and hazard ratio (HR), respectively, along with the 95% confidence interval (CI) and *p*-values.

## 3. Results

Of a total of 529 IE patients, 128 (24.2%) had IE mediated by *S. aureus*. Patients with SA-IE were mostly male (69.3%) but had a higher proportion of females compared to non-SA-IE patients (30.7% vs. 21.8%; *p* = 0.039) and were significantly younger (61.1 [45.8–69.9] vs. 66.1 [54.3–74.4]; *p* = 0.002). Patients with SA-IE had comparable comorbidities to patients with non-SA-IE. Regarding risk factors, SA-IEs were associated with more active hepatitis (14.1% vs. 4.2% *p* < 0.001), HIV (6.3% vs. 1% *p* = 0.001), alcohol abuse (13.3% vs. 7.2% *p* = 0.034), and i.v. drug abuse (10.9% vs. 5% *p* = 0.017). In addition, SA-IEs patients suffered from more pronounced symptoms with more fever occurrences (68.8% vs. 56.9% *p* = 0.017), a higher rate of preoperative sepsis (68% vs. 37.2% *p* < 0.001, preoperative neurological symptoms (42.2% vs. 22.9% *p* < 0.001), and septic embolism (41.4% vs. 29% *p* = 0.009). Patients with SA-IE were shown to experience more frequent hemodynamic instability (21.1% vs. 10.2% *p* = 0.001) and were more likely to be intubated before surgery (26.6% vs. 11.7% *p* < 0.001) (Table 1).

**Figure 1:**
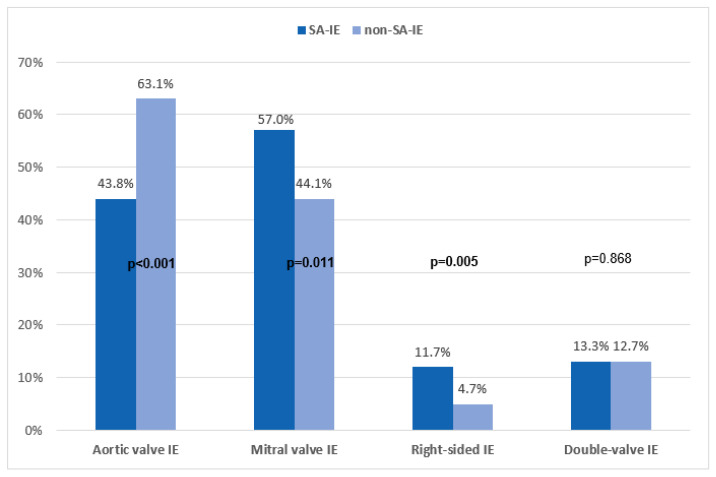
Distribution of valve involvement depending on the causative agent; SA-IE (*n* = 128) vs. non-SA-IE (*n* = 401). SA-IE = *Staphylococcus aureus* infective endocarditis, non-SA-IE = IE caused by agents other than *Staphylococcus aureus*.

Looking at the echocardiographic data, it is striking that SA-IE was associated with more vegetations compared to non-SA-IE (89.6% vs. 76.6%; *p* = 0.002). SA-IE is significantly more likely to involve the mitral valve (57% vs. 44.1%; *p* = 0.011) and the tricuspid valve (10.9% vs. 4.2%; *p* = 0.005) (Table 2). Aortic valve involvement is more likely in non-SA-IE (43.8% vs. 63.1%; *p* < 0.001) (Figure 1). The number of combined procedures, as well as operative times including bypass and cross-clamp times, were similar in both groups. Operative data are listed in the (Supplementary Table S1).

**Figure 2:**
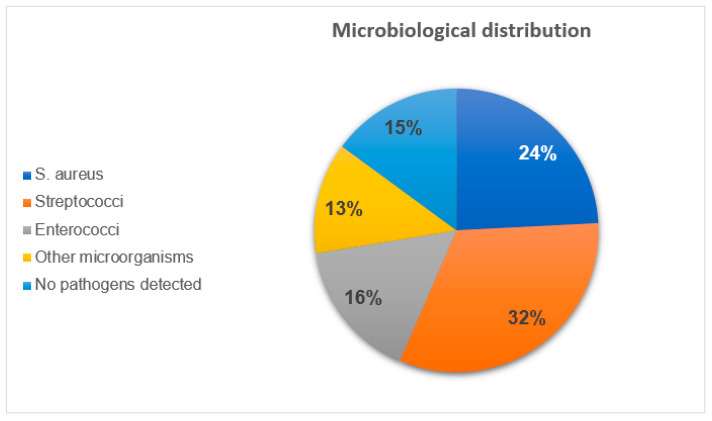
Microbiological distribution (*n* = 529).

In the examined population, Streptococci were the most frequently detected microorganism with 32.3%, followed by *S. aureus* with 24.2%, and enterococci with 15.7% (Figure 2). MRSA infections were documented in 2.1% of the entire population, equivalent to 8.6% of the SA-IE group. Mixed infections were documented in 7.7% of cases.

Regarding postoperative outcome, we found a significantly higher 30-day mortality after cardiac surgery in patients with SA-IE (20.3% vs 12.5%; *p* = 0.028). However, the 1-year mortality rate did not differ (29.4% vs. 22.2%; *p* = 0.121). Furthermore, patients with SA-IE had a longer stay in the intensive care unit (5 [2–10] vs. 6 [3–12]; *p* = 0.003), required longer ventilation (40% vs. 20.8%; *p* = 0.047), and received tracheotomy more frequently (25.2% vs. 13.5%; *p* = 0.002). There were no relevant differences in other complications (Table 3).

**Figure 3:**
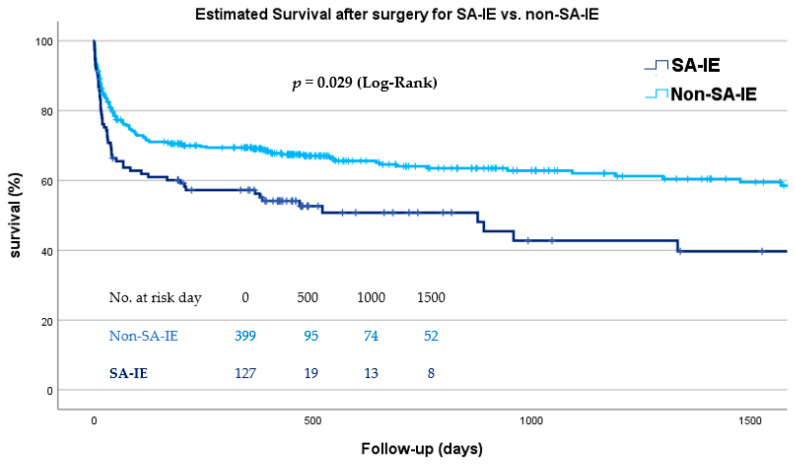
Cumulative survival after surgery for infective endocarditis according to causative agent; SA-IE vs. non-SA-IE. SA-IE = *Staphylococcus aureus* infective endocarditis, non-SA-IE = IE caused by agents other than *Staphylococcus aureus*.

Cumulative survival was analyzed using a Kaplan–Meier survival function for both groups, SA-IE and non-SA-IE. Patients with SA-IE had significantly lower long-term survival compared to non-SA-IE, as shown in Figure 3 (log-rank *p* = 0.029). The median survival for patients with SA-IE is 876 days with a 95% confidence interval of (379–1373) days. In non-SA-IE, median survival times cannot be provided, since less than half of patients died within the follow-up period. The median follow-up was 3.6 years [IQR 1.82–4.83]. To allow for comparability, survival probabilities are provided as follows in Table 4.

In the logistic regression analysis, staphylococcal infection was identified as an independent risk factor of 30-day mortality, with OR 1.827 [1.1127–2.963]; *p*= 0.015, as well as for 1-year mortality, HR 1.648 [1.074–2.528]; *p*= 0.022. Other risk factors for mortality, including advanced age, perivalvular abscess, and preoperative renal insufficiency, have previously been described [10].

In the examined cohort, 146 (27.6%) patients suffered from neurological complications preoperatively. Preoperative neurological complications were significantly more prevalent in SA-IE compared to non-SA-IE group with 42.2% vs. 22.9%; (*p* < 0.001). Ischemic insults were the most prevalent in both groups with 20.3% vs. 12.2%, respectively (*p* < 0.022). Preoperative TIA or cerebral bleeding did not vary between groups (Table 5).

**Figure 4:**
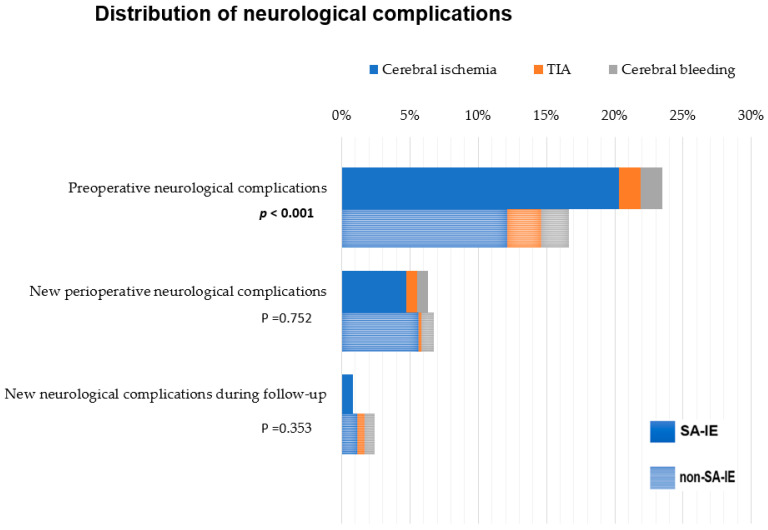
Distribution of neurological complications in the SA-IE (*n* = 128) vs. non-SA-IE (*n* = 401): TIA = transient ischemic attack, SA-IE = *Staphylococcus aureus* infective endocarditis, non-SA-IE = IE caused by agents other than *Staphylococcus aureus*.

Postoperatively, new neurological complications were documented in 6.0% of all patients. There were no significant differences in neurological complications of any kind between patients with an SA-IE and patients with a non-SA-IE (Figure 4). Regarding log-term follow-up, we did not find any significant differences between patients with SA-IE and patients with non-SA-IE. Median duration of the follow-up was 3.6 years [IQR 1.82–4.83] with 156 patients included.

## 4. Discussion

In this study, we present a thorough analysis of patients diagnosed with IE, with a specific focus on cases mediated by *Staphylococcus aureus*, and investigate the concomitant neurological complications in individuals undergoing valve surgery. The key findings of our investigation are summarized as follows:

(i) Patients with SA-IE were younger and had comparable comorbidities, yet they suffered severe clinical manifestation characterized by higher prevalence of fever, preoperative hemodynamic instability, and mitral valve involvement.
(ii) Both short-term (30-day) and long-term survival were significantly poorer in patients with SA-IE compared to other IE patients.
(iii) Despite a higher frequency of preoperative neurological complications in the SA-IE group, the incidence of peri- and postoperative new onset neurological complications were comparable between both groups.

In clinical manifestations, we observe a younger age group being affected by SA-IE. The prominence of a younger age group in *S. aureus* endocarditis may point towards specific risk factors or modes of transmission that differ from those commonly attributed to endocarditis in general like i.v. drug usage which is associated with younger age [12]. However, the small amount of people with i.v. drug usage cannot explain the gap in age. Another aspect which was shown in our registry is the fact that patients who suffer from SA-IE are less likely to have preexisting valvular pathologies. The explanation for this could lie in *S. aureus* itself. *S. aureus* has many pathogenic factors which allow it to also attack healthy endothelium [4]. This can lead to IE in younger patients with less predisposing factors if they have an *S. aureus* blood stream infection. Nevertheless, further investigations are warranted to elucidate the underlying reasons for this age-related predilection and its implications for disease management and prevention.

The distribution of sex observed in the present study, in which a higher proportion of females were represented among SA-IE cases, despite constituting only a quarter of the total study population, has been reported in other studies [9,13]. This prompts a reconsideration of risk factors and potential sex-specific vulnerabilities that may contribute to the pathogenesis of SA-IE.

The predilection of SA-IE for the mitral valve is an intriguing aspect of our findings. This finding was also reported in a Swedish register study from 2019 [14]. While mitral valve involvement is not uncommon in IE, the heightened frequency observed in SA-IE cases raises questions about the organism’s specific tropism for this valve. Gaining a clearer understanding of the molecular and pathophysiological factors that cause *S. aureus* to preferentially affect the mitral valve could offer valuable insights into disease progression and support the development of more targeted therapeutic strategies.

SA-IE had a higher 30-day mortality rate compared to non-SA-IE (20.3% vs. 12.5%; *p* = 0.028). These findings are in line with the results of previous studies, which indicate that approximately one in five patients with SA-IE die within the first 30 postoperative days [15,16]. A plausible explanation is probably the aggressive nature of Staphylococcal IE with higher prevalence of septic embolic events and hemodynamic instability [16]. Embolic events have been shown to be independently associated with worse outcomes [17].

Moreover, the Kaplan–Meier analysis visually demonstrates a significantly poorer long-term survival among patients with SA-IE (*p* = 0.029). This observation aligns with the results of a large-scale Danish study associating SA-IE with the highest in-hospital as well as long-term mortality rates [18].

Our analysis demonstrated a markedly higher incidence of neurological complications in SA-IE at 42.2%, as opposed to the 22.9% observed in non-SA-IE cases (*p* < 0.001). It has previously been reported that *S. aureus* is a risk factor for neurological complications in IE [19]. This may be attributed to the inherent characteristics of *S. aureus* as a virulent agent. *S. aureus* possesses the propensity for larger vegetation, which increases the susceptibility to neurological complications [3]. Our analysis showed that patients with SA-IE had significantly more vegetations, at 87.5%, compared to 76.1% in the non-SA group (*p* = 0.002). In vitro models support the notion that microorganism attachment is often preceded by aseptic endothelial damage or inflammation within the endothelium. This can result in sterile vegetation, acting as the focal point for endocarditis in the context of bacteremia. *S. aureus*, with its multitude of pathogenicity factors, exhibits a unique ability to colonize damaged endothelium by binding von Willebrand factor (vWF)-adhesin molecules. Additionally, *S. aureus* demonstrates the capacity to modulate the immune system and form biofilms [3]. Further understanding of the pathogenesis of vegetation development and persistence could provide an approach for targeted treatments that prevent embolic complications and improve clinical outcomes in such conditions.

A noteworthy observation in the sub-analysis is the significantly higher incidence of ischemic events in patients with SA-IE. In the examined population, postoperative transient ischemic attacks (TIAs) and hemorrhagic cerebral bleeding were infrequent, occurring in only 2.4% and 1.7% of patients, respectively. *S. aureus* has previously been identified as a risk factor for both embolic and hemorrhagic cerebral complications [19,20]. However, our analysis showed no significant difference between the SA-IE group and the non-SA-IE group concerning hemorrhagic cerebral insults, possibly due to the low number of incidents in our study.

A distinct pattern emerged with regard to postoperative neurological complications. SA-IE did not exhibit a higher risk for neurological complications after valve surgery, suggesting that surgery in SA-IE may contribute to a reduction in the risk of such complications. This trend persisted in the long-term follow-up, where no significant differences were observed between patients with SA-IE and those with non-SA-IE. Similar to the findings in our study, a Spanish multicenter study from 2013 reported that patients with *S. aureus* endocarditis were two to three times more likely to experience neurological complications [19]. Antimicrobial therapy was shown to decrease the risk after one week; however, for patients with substantial vegetations, as frequently observed in SA-IE, the risk for neurological complications remained high. A differentiation between conservative and surgical management was not conducted in that study [19]. Other studies have identified the benefits of early surgery in preventing embolic complications and lowering mortality [21,22].

### Study Limitations

Our study has several limitations that should be considered when interpreting the results. Firstly, the retrospective nature of our data collection process introduces the possibility of losing relevant information, potentially affecting the overall completeness of our dataset. Unfortunately, it was not possible to conduct a sub-analysis to compare MSSA and MRSA due to the low number of MRSA-mediated IE cases.

Given our hospital’s role as a reference center for cardiac surgery, a considerable number of patients were transferred with indications for surgery. This circumstance could lead to an overrepresentation of patients experiencing neurological incidents, potentially influencing the observed outcomes.

Furthermore, the transfer of patients raises concerns about the potential loss of externally diagnosed neurological events during the process.

## 5. Conclusions

In conclusion, our study highlights a significant disparity in the incidence of neu-rological complications between patients with SA-IE and those with non-SA-IE. Patients afflicted with SA-IE exhibited a notably higher prevalence of neurological complications compared to non-SA-IE. However, following valve surgery, we observed a remarkable decrease in neurological complications across both patient cohorts, with no discernible difference in the occurrence of neurological complications between patients with SA-IE and those with non-SA-IE. This suggests that valve surgery effectively reduces the risk of neurological complications associated with SA-IE, aligning the postsurgical outcomes between the two groups. The lack of disparity in neurological complications after surgery suggests that earlier surgical intervention in patients with SA-IE may help to reduce neurological sequelae and improve overall prognosis. Further research should focus on defining optimal surgical timing to improve outcomes and reduce the burden of complications in such patients.

## Figures and Tables

**Table 1:** Patient demographics and preoperative characteristics.

	All Patients (*n* = 529)	SA-IE (*n* = 128)	Non-SA-IE (*n* = 401)	*p*-Value
**Demographics**				
Age (years)	65.12 [51.65–73.64]	61.12 [45.83–69.92]	66.10 [54.27–74.42]	**0.002**
Female sex	126 (23.9%)	39 (30.7%)	87 (21.8%)	**0.039**
BMI (kg/m^2^)	25.51 [23.36–28.41]	26.16 [23.88–29.86]	25.39 [23.12–28.01]	**0.031**
LVEF < 30%	17 (3.2%)	5 (3.9%)	12 (2.9%)	0.711
Logistic EuroSCORE (%)	10.21 [4.68–22.03]	9.69 [4.73–19.26]	10.75 [4.68–23.08]	0.735
EuroSCORE II (%)	8 [5–11]	8 [5.5–10]	8 [5–11]	0.716
**Preoperative data**				
Hypertension	343 (64.8%)	80 (62.5%)	263 (65.6%)	0.524
Hyperlipidemia	158 (29.9%)	32 (25%)	126 (31.4%)	0.167
Coronary heart disease	149 (28.2%)	38 (29.7%)	111 (27.7%)	0.660
Cerebrovascular disease	66 (12.5%)	53 (13.2%)	13 (10.2%)	0.362
Diabetes mellitus	136 (25.7%)	30 (23.4%)	106 (26.4%)	0.499
Peripheral artery disease	48 (9.1%)	9 (7%)	39 (9.7%)	0.355
COPD	56 (10.6%)	13 (10.2%)	43 (10.7%)	0.856
Active smocking	113 (21.4%)	29 (22.7%)	84 (20.9%)	0.681
Pulmonary hypertension	51 (9.6%)	10 (7.8%)	41 (10.2%)	0.421
Renal insufficiency	289 (54.6%)	77 (60.2%)	212 (52.9%)	0.149
Leucocytes/µL	10.2 ± 6.8	12 ± 6.5	9.7 ± 6.8	**<0.001**
CRP (mg/L)	42.5 [9.5–120.7]	124.2 [70.2–230.7]	51.7 [7.4–81.4]	**0.003**
Procalcitonin (µg/L)	72.7 ± 39.2	15.0 ± 21.2	10.3 ± 13.1	0.817
**Risk Factors**				
Previous history of IE	31 (5.9%)	4 (3.1%)	27 (6.7%)	0.130
Underlying valve dysfunction	185 (35%)	35 (27.3%)	150 (37.4%)	**0.038**
Mitral valve prolapse	30 (5.7%)	5 (3.9%)	25 (6.2%)	0.321
Congenital heart anomaly	15 (2.8%)	5 (3.9%)	10 (2.5%)	0.402
Prosthetic valve	117 (22.1%)	24 (18.8%)	93 (23.2%)	0.292
Immune suppression	9 (1.7%)	3 (2.3%)	6 (1.5%)	0.519
HIV	12 (2.3%)	8 (6.3%)	4 (1%)	**0.001**
Active malignancy	50 (9.5%)	9 (7.0%)	41 (10.2%)	0.282
Alcohol abuse	46 (8.7%)	17 (13.3%)	29 (7.2%)	**0.034**
Intra-venous drug abuse	34 (6.4%)	14 (10.9%)	20 (5%)	0.017
Active hepatitis	35 (6.6%)	18 (14.1%)	17 (4.2%)	**<0.001**
**Symptoms**				
Fever	316 (59.7%)	88 (68.8%)	228 (56.9%)	**0.017**
Sepsis	236 (44.6%)	87 (68%)	149 (37.2%)	**<0.001**
Neurological symptoms	146 (27.6%)	54 (42.2%)	92 (22.9%)	**<0.001**
Hemodynamic instability	68 (12.9%)	27 (21.1%)	41 (10.2%)	**0.001**
IABP preoperatively	3 (0.6%)	3 (2.3%)	0 (0%)	**0.002**
Preoperative intubation	81 (15.3%)	34 (26.6%)	47 (11.7%)	**<0.001**
Septic embolism	168 (32.1%)	53 (41.4%)	115 (29.0%)	**0.009**
Myocardial infarction	13 (2.5%)	10 (2.5%)	3 (2.3%)	0.924

SA-IE = *Staphylococcus aureus* infective endocarditis, non-SA-IE = IE caused by agents other than *Staphylococcus aureus*, BMI = body mass index, CRP = C-reactive protein, COPD = chronic obstructive pulmonary diseases, ECMO = extra corporal membrane oxygenator, HIV = human immunodeficiency virus, HLM = heart lung machine, IABP = intra-aortic balloon pump, **Bold** indicates *p* < 0.05.

**Table 2:** Valvular manifestation.

	All Patients (*n* = 529)	SA-IE (*n* = 128)	Non-SA-IE (*n* = 401)	*p*-Value
Left–sided IE	501 (94.7%)	118 (92.2%)	383 (95.5%)	0.144
AVE	309 (58.4%)	56 (43.8%)	253 (63.1%)	**<0.001**
MVE	250 (47.3%)	73 (57.0%)	177 (44.1%)	**0.011**
Right–sided IE	34 (6.4%)	15 (11.7%)	19 (4.7%)	**0.005**
TVE	31 (5.9%)	14 (10.9%)	17 (4.2%)	**0.005**
PVE	3 (0.6%)	1 (0.8%)	2 (0.5%)	0.711
Double-valve endocarditis	68 (12.9%)	17 (13.3%)	51 (12.7%)	0.868
Vegetations	417 (78.8%)	112 (87.5%)	305 (76.1%)	**0.002**
Paravalvular involvement	222 (42.0%)	51 (39.8%)	171 (42.6%)	0.576
abscess	160 (30.2%)	38 (29.7%)	122 (30.4%)	0.875
fistula	15 (2.8%)	3 (2.3%)	12 (3.0%)	0.700
perforation	106 (20.0%)	27 (21.1%)	79 (19.7%)	0.732

SA-IE = *Staphylococcus aureus* infective endocarditis, non-SA-IE = IE caused by agents other than *Staphylococcus aureus*, AVE = aortic valve endocarditis, MVE = mitral valve endocarditis, PVE = pulmonary valve endocarditis, TVE = tricuspid valve endocarditis, Bold indicates *p* < 0.05.

**Table 3:** Postoperative outcomes.

	All Patients (*n* = 529)	SA-IE (*n* = 128)	Non-SA-IE (*n* = 401)	*p*-Value
30-day mortality	76 (14.4%)	26 (20.3%)	50 (12.5%)	**0.028**
1-year mortality	126 (23.8%)	37 (29.4%)	89 (22.2%)	0.121
Re-thoracotomy	83 (15.7%)	18 (14.2%)	65 (16.3%)	0.576
Tracheotomy	86 (16.3%)	32 (25.2%)	54 (13.5%)	**0.002**
Pacemaker implantation	51 (9.7%)	11 (8.6%)	40 (10%)	0.639
Myocardial infarction	3 (0.6%)	2 (1.6%)	1 (0.3%)	0.087
PCI	2 (0.4%)	0 (0%)	2 (0.4%)	0.424
Neurological event	32 (6.1%)	7 (5.5%)	25 (6.3%)	0.757
Acute renal failure	177 (33.6%)	48 (37.8%)	129 (32.3%)	0.249
Ventilation duration (hours)	22 [11.8–117.32]	40 [12.76–170.50]	20.8 [11.53–96.75]	**0.047**
ICU stay (days)	5 [2–10]	6 [3–12]	4 [2–9]	**0.003**
In-hospital stay (days)	12 [9–18]	13 [8.5–19]	12 [9–17.75]	0.965

SA-IE = *Staphylococcus aureus* infective endocarditis, non-SA-IE: IE caused by agents other than *Staphylococcus aureus*, ICU = intensive care unit, PCI = percutaneous coronary intervention, Bold indicates *p* < 0.05.

**Table 4:** Survival probabilities.

Survival Probability	SA-IE	Non-SA-IE
1 year	57% (95% CI: 45–69)	69% (95% CI: 59–79)
3 year	42% (95% CI: 30–54)	62% (95% CI: 50–74)
5 year	35% (95% CI: 23–47)	56% (95% CI: 42–70)

**Table 5:** Neurological complications sub-analysis.

	All Patients (*n* = 529)	SA-IE (*n* = 128)	Non-SA-IE (*n* = 401)	*p*-Value
**Preoperative neurological complications**	146 (27.6%)	54 (42.2%)	92 (22.9%)	**<0.001**
Ischemia	75 (14.2%)	26 (20.3%)	49 (12.2%)	**<0.022**
TIA	12 (2.6%)	2 (1.6%)	10 (2.5%)	0.380
Cerebral bleeding	10 (1.9%)	2 (1.6%)	8 (2.0%)	0.754
**New perioperative neurological complications**	32 (6.0%)	7 (5.5%)	25 (6.2%)	0.752
Ischemia	26 (4.9%)	6 (4.7%)	20 (5.9%)	0.891
TIA	2 (0.4%)	1 (0.8%)	1 (0.2%)	0.393
Cerebral bleeding	5 (0.9%)	1 (0.8%)	4 (0.9%)	0.826
**Neurological complications during follow-up**	11 (2.1%)	1 (0.8%)	10 (2.5%)	0.353
Ischemia	6 (1.1%)	1 (0.8%)	5 (1.2%)	0.841
TIA	2 (0.4%)	0 (0.0%)	2 (0.5%)	0.478
Cerebral bleeding	3 (0.7%)	0 (0.0%)	3 (0.8%)	0.384

SA-IE = *Staphylococcus aureus* infective endocarditis, non-SA-IE = IE caused by agents other than *Staphylococcus aureus*, TIA = transient ischemic attack, Bold indicates *p* < 0.05.

## Data Availability

The datasets generated and analyzed during the current study are available from the corresponding author on reasonable request.

## Supplementary Materials

The following supporting information can be downloaded at https://www.mdpi.com/article/10.3390/jcm14197114/s1, Table S1: Operative data.
