# Supplementary material for: Surgery in Staphylococcus aureus Infective Endocarditis: Clinical Outcomes, Neurological Sequelae, and Prognostic Implications
Source: J Clin Med. 2025 Oct 9;14(19):7114. doi: 10.3390/jcm14197114 (PMC12524898; doi:10.3390/jcm14197114)
Supplement: Supplementary file 1 [file jcm-14-07114-s001.zip › jcm-3891496-supplementary.pdf]

Supplementary Table S1: operative data

	**All patients (n=529)**	**SA-IE (n=128)**	**Non-SA-IE (n=401)**	**p-value**
Days between diagnosis and surgery	8 [4-18]	7 [3-14]	9 [4-20]	0.085
Combined procedures	215 (41.2%)	52 (41.3%)	163 (41.2%)	0.983
Operative time in minutes	205 [159-262]	203 [158.25-265.25]	205 [159.25-261]	0.828
Bypass time in minutes	116 [86.25-154]	109.5 [83-153.75]	116.5 [87-154.75]	0.463
Cross clamp time in minutes	75.5 [56-100]	68.5 [51-100.25]	77 [57-100]	0.179
Hemofiltration on HLM	48 (29.4%)	14 (33.3%)	34 (28.1%)	0.521
MCS (ECMO and/or IABP	33 (6.2%)	10 (7.8%)	23 (5.7%)	0.398

SA-IE = Staphylococcus aureus infective endocarditis, non-SA-IE = IE caused by agents other than Staphylococcus aureus, ECMO = extra corporal membrane oxygenator, HLM = heart lung machine, IABP = intra-aortic balloon pump.
